# Examining the effect of land transfer on landlords' income in China: An application of the endogenous switching model

**DOI:** 10.1016/j.heliyon.2020.e05071

**Published:** 2020-10-03

**Authors:** Thomas Bilaliib Udimal, E Liu, Mingcan Luo, Ya Li

**Affiliations:** College of Economics and Management, Southwest Forestry University, Kunming Yunnan Province 650224, PR China

**Keywords:** Farmland, Land transfer, Out-migration, China, Agricultural economics, Agricultural policy, Business, Economics, Sociology

## Abstract

The paper looks at factors that influence landlords' decision to transfer their farmlands and how farmland transfer has impacted on landlords' income. The essence of farmland transfer policy is to promote efficiency in agricultural land use. Endogenous regression model was adopted for the study because of its ability to handle the transfer decisions and impact of transfer decision on outcome simultaneously. The data were obtained from selected communities in Yunnan province, China. A total of 260 landlords were randomly selected for the study. The result shows that out-migration, off-farm income, agesq, public infrastructure and skill training influence transfer decision positively. The results further show that famine experience, access to credit, education and age negatively influence farmland transfer decision. The results show that farmland transfer leads to a significant increase in landlords' income.

## 1 Introduction

The reforms in China have led to a rapid transformation and changes in the structure of the economy from a centralised economy to a market led economy (Li et al., 2015). The reforms have led to an increase in non-farm land use due to increase in the demand for land for non-agricultural activities (Su et al., 2018). It is estimated that China has a total land area of about 960 million hectares (Mha), but only 14.8% is used for the cultivation of food crops and horticultural products (Fan et al., 2013; Qiang et al., 2013). China can only boast of one-third of global arable land. With already limited arable land, China stands a risk of losing its already limited arable land to faster growing industrial sector (Rigg et al., 2009). To safeguard the food supply in the country, the government in its 11th Five-Plan has put in place a policy to ensure that arable land does not fall below 120 million hectares (Mha). The decreasing land size has affected the farmers' ability to modernize their agricultural activities to ensure efficiency of their operations, as they cannot adopt the use of machinery due to limited land size. The development has affected the commercialization of agriculture and led to a reduction in farmers' income.

The average farmland under China's household responsibility system (HRS) is approximately 0.5 ha per household (Ni, 2015). The development of other sectors of economy as a result of rapid industrialization has led to many leaving farming for non-farm jobs (Li, 2013). The expansions in other sectors have led to abandonment of farmlands especially in rural China (Liu et al., 2014). In an attempt to promote efficiency in arable land usage and improve landlords' income, the government has put in place a policy to ensure smooth farmland transfer from landlords to tenants who have the capabilities to ensure its efficient use. The policy is geared towards agricultural development and modernization. The development has led to the acquisition of farmlands by managers, agricultural companies, and cooperatives to improve the efficiency of land usage by introducing modern managerial system into the sector.

Land transfer policy is a major intervention by the central government to inject efficiency in agricultural land use, and modernization of agriculture sector. The policy seeks to take farmlands from less efficiency producers to efficient producers through contractual agreements. The policy has since suffered some setbacks due to some landlords unwilling to transfer farmlands to cooperatives (Su et al., 2018). Some migrant workers still hold on to their farmlands and are unwilling to transfer (Zuo et al., 2015). The development is said to have limited the scope of operation of cooperatives and other agricultural companies who are ready to inject much capital into the sector (Wang et al., 2015). For the policy on China's farmland transfer to achieve its objective of improving efficiency in the agricultural sector and revitalizing rural life, there is the need to assess the influence of economic and non-economic factors on the landlords' decision to transfer farmland.

Some studies have looked at the determinants of land transfer across different provinces in China (Yan and Huo, 2016; Gao et al., 2014). The study by Chen et al. (2010) in Jiangxi province concluded that household members' migration has a positive effect on farmland transfer among rural households. The research on rent-out and rent-in decision by households concludes that availability of off-farm activities promotes rent-out as against rent-in (Huang et al., 2012). Che (2016b) having household members as migrant workers or participating in off-farm income activities increase the tendency of rent-out but less likely to engage in rent-in activities. The study by Su et al. (2018) concluded that stability of off-farm employment influences the transfer of farmland. The study further noted that willingness to stay in the city plays a complementary role in farmland transfer. However, other studies' findings oppose the findings that off-farm income activities promote farmland transfer. The study by You and Wu (2010) concluded that off-farm income has no impact on land transfer decision. Su et al. (2018) argued that the decision to transfer farmland is not grounded on economic factors alone but includes social and psychological wellbeing of the landowners.

Even though previous studies have looked at factors influencing farmlands transfer, none has tried to establish the link between land transfer and landlords' income. The essence of land transfer is to inject efficiency and also improve income levels of landlords. It is therefore, imperative to look at the impact of farmlands transfer on the income levels of the landlords. This study seeks to examine factors that influence farmland transfer and how farmlands transfer has impacted on landlords' income.

On the methodological approach, extant literature has looked at the impact of adoption decision on farm income (Bravo-Ureta et al., 2006; Posthumus et al., 2010; Kassie et al., 2011; Amare et al., 2012). According to Bravo-Ureta et al. (2006), there exist a positive significant relationship between agro-forestry systems adoption and farm income. Kassie et al. (2011), through the use of propensity score matching (PSM) found a significant relationship between the use of improved groundnut varieties and crop income. There are challenges associated with the use PSM because of its unconfoundness assumption. According to this assumption once observable characteristics are controlled, adoption process becomes random and uncorrelated with the outcome variables. Smith and Todd (2005) noted the differences in the outcomes between the adopters and non-adopters as selections are based on characteristics that cannot be measured. The study by Abdulai and Huffman (2014) on the adoption and impact of soil and water conservation technology applied endogenous switching model. They concluded that the adoption of soil and water conservation technology leads to an improved rice yields and net return. Apart from the methodological difference, this study looks the impact of farmland transfer on landlords' income, the dimension that has not been considered in previous researches.

In this study, endogenous switching regression model as applied by Abdulai and Huffman (2014) is adopted for application.

## 2 Farmland transfer reforms in China

The farmland transfer in China can be traced back to the development in Xiaogang a village in Anhui province where 18 farmers took a decision to allocate their collectively owned farmland to individual households. Through its experimentation in subsequent years the HRS was established leading to farmland contracting rights system in rural China (Lin, 1988). Rural households have the right to the use of the land apportioned to them by the rural collective. However, rural collective still holds the legal title of the land. Households are not allowed to use the land apportioned to them as collaterals in acquisition of loans. The challenges associated with the management of spatially allocated farmlands led to transfer of farmlands. The process was however illegal, as households were not given the right to transfer farmlands allocated to them. The process was not organized as there was not an established unit to coordinate their activities (Su et al., 2018). The process to formalized farmland transfer in China started in 1984, where it was captured in the 'No.1 Document' of the Central Committee of the Communist Party of China. It was agreed that households be allowed to transfer their farmlands to other households but on condition that permission is obtained from rural collectives (Chen et al., 2010). The development led to a complete paradigm shift in farmland transfer in 1986, where government started encouraging transfer of farmlands to households who have managerial skills and ability to boost production. Subsequent years led to initiation of various policies for improvement of smallholders. A series of reforms in farmland transfer has resulted to the use of farmland as collateral for loans, land transfer and exchange (Jin and Deininger, 2009). To ensure that benefits due households are realized; in 2003, Contracting Law was promulgated to secure the interest of tenants. The right for rural households to go into contractual agreement regarding the use of land was officially given recognition under Property Right Law of 2007. To help deal with land disputes and protect land rights in the rural area, Arbitration Law of Rural Contracted Land Disputes was established in 2008. To ensure compliance with the enacted laws, land rights confirmation, registration and certification was implemented on trial basis in 2009. Twenty-eight provinces have so far implemented the current model, which started initially with three provinces. These developments finally led to distinct land rights in China comprising ownership right, contract right and management right (Su et al., 2018).

## 3 Data

The data for the study were collected through a field survey between September to December 2019 in the selected communities in Yunnan Province. Prior to the field survey, land transfer secretariats in the respective communities were contacted for information on the presence of tenants farmers. Land transfer secretariats assisted with the list of landlords for both transferred and those who did not transfer their farmlands in the respective communities. Ten communities were purposively selected based on the presence of both transferred landlords and landlords who did not transfer their farmlands. Systematic sampling technique was used to select the required sample for the study. Thirty landlords consisting both transferred and those who did not transfer were sampled from each community as there were not much differences in terms of landlords population. However, due to absence of some landlords, the required number of thirty landlords for each community was not met in some communities. As a result, two hundred and sixty (260) landlords took part in the study instead of three hundred (300). Information from the landlords was obtained through face to face interviews with the help of questionnaires. There was forward and backward translation of the questionnaire as it was initially translated into Chinese and later translated back into English. The interviews were conducted in Chinese. Enumerators who could speak both Chinese and English were engaged for the data collection. The information gathered included landlords' socioeconomic characteristics and land characteristics. Additional information was also sourced from land transfer secretariats in the respective communities. One hundred and sixty-three (163) landlords representing 62.7% landlords who have transferred their farmlands and ninety-seven (97) representing 37.3% for landlords who did not transferred their farmlands took part in the study.

### 3.1 Conceptual framework

The transfer of farmland is modeled with the assumption that landlords have the option to transfer or not to transfer. This is based on the assumption that landlords are risk neutral, and will factor in the benefit they will derive from the transfer of their farmlands. It is presumed that landlords will opt for the option that will give them maximum utility. The benefit a landlord derives from the transfer of farmland is denoted by Υ_jT_ and the benefit for refusal to transfer is denoted by Υ_jN_. The benefit is the income landlords derive.

The two scenarios/regimes are represented as:

(1) Υ_jT_ = Χ_j_β_T_ + μ_jT_

and

(2) Υ_jT_ = Χ_j_β_N_ + μ_jN_

where *X*_*j*_ is a vector of socio-economic, land and household characteristics;*B*_*T*_ and *B*_*N*_ are vectors of parameters; μ_jT_ and μ_jT_ are iids. The landlord will transfer his/her farmland if the net benefit to be obtained is higher than when the farmland is not transferred thus, Y_jT_ > Y_jN_ (Pitt, 1983; Abdulai and Huffman, 2014). Even though the preferences of landlords, thus the perceived net benefits of farmland transfer, are unknown, the characteristics of landlords are observable.

The net benefit derived from the transfer of farmland is denoted by a latent variable D^'^_j_, though not observable but expressed as a function with observable attributes and characteristics represented by Ζ in the Equation as:

(3) D_j_∗ = γ^'^ Ζ_j_ + ε_j_, D_j_ = 1[D_j_∗>0],

where D_j_ is a binary variable that equals 1 for landlords who transferred their farmlands, and zero for otherwise. The vector of parameter to be estimated is denoted by γ. The landlords will only transfer their farmlands if they are convinced of higher returns from the transfer. The error term ε and variance σ^2^_ε_ capture the measurement errors and unobserved factors but possibly known to the landlords. The Ζ term represents factors that influence landlords farmland transfer decision (Abdulai and Huffman, 2014).

### 3.2 Empirical model specification

The decision by individuals to either transfer or not transfer their farmlands is based on the benefits the stand to gain. The transfer of farmlands by the landlords needs to be factored in when examining the outcomes such as income generation. Absence of such consideration will lead to selection bias. This is because landlords who perceive that they would obtain less income from the land transfer, may choose not to transfer their farmlands and that will truncate the observed land transfer benefit distribution (Pitt, 1983; Abdulai and Huffman, 2014). The issue of selection bias can also arise if unobservable factors influence the respective error terms in the land transfer choice equation (ε) and the outcome equation (μ), thus leading to correlation of error terms in the land transfer choice equation and outcome equation, with (ε, μ) = ρ.

The unobservable factors that could lead to the correlation in the error terms in the two equations can be the transaction cost landlords may have to incur in the process of transferring their farmlands, their understanding of issues relating to land transfer policy, and the social network of landlords as the people they come into contact with may either influence their decision to transfer or not to transfer. According to Suri (2011), prior knowledge of such constraints can enable policy makers to formulate policies to limit their potential impact on land transfer. Failure to capture unobservable factors, will lead to a correlation between the independent variables and the error term leading to ρ ≠ 0. Under such circumstances, OLS would produce biased results.

In analysing the effect of land transfer on landlords' income, it is quiet challenging to attribute the differences in income between the transferred landlords and non-transferred landlords to the transferred decision. According to Miguel and Kremer (2004), when experimental data through randomized trials are available, it is easy to deal with the issue of causal inference with the information on counterfactual. With cross-sectional data as in the case of the current study, there is no information on the counterfactual. To help deal with this shortcoming associated with cross-sectional data (Dehejia and Wahba, 2002), argued that the direct effect of technology adoption should be analysed by looking at the differences in outcomes among farm households.

Rosenbaum and Rubin (1983) proposed propensity score matching approach for the study of impacts of technology adoption on farm outcomes and household welfare, particularly when self-selection is an issue (Amare et al., 2012). However, the main aim of propensity score matching is to draw a balance of the observed distribution of covariates across the groups of transferred and non transferred landlords. Logit and probit models have been used extensively in studies that involve binary choice. However, they are not applicable in the current study. The current study apart from considering the determinants of land transfer that is a binary choice it goes further to look at the impact of land transfer on landlords' income. To help achieve this objective endogenous switching regression model is used. The choice of endogenous switching regression model is due to its ability in handling selection bias that is inherent in the estimation of the impact of adoption on outcomes.

Lee (1982) came out with an endogenous switching regression model and used it to generalize Heckman's selection correction approach. It handles the selection on unobservable as it treats selectivity as an omitted variable problem (Abdulai and Huffman, 2014). Endogenous switching classifies landlords into transferred and non-transferred in order to capture their differential response.

Given that the landlords may either transfer or not transfer, the observed outcome takes a form of:

Regime 0 (non-transferred): Υ_jN_ = X^'^β_jN_ + μ_jN_ if D_j_ = 0

(4) Regime 1 (transferred): Υ_jT_ = X^'^ β_jT_ + μ_jT_ if D_j_ = 1

where Υ_jT_ and Υ_jN_ = are the outcome variables for the transferred and non-transferred, respectively, X′ is a vector of fixed factors and factor prices, and household characteristics. The vectors β and γ in Eqs. (4) and (3), respectively are the associated parameters to be determined. It is possible for the variables in the vectors X in Eq. (4) and Ζ in Eq. (3) to overlap, but there must be at least one variable in Ζ that does not appear in the X.

There is a possibility of nonzero variances between the error terms of transfer decision and outcomes if self-selection into transfer and non-transfer approach is adopted. The error terms ε, μ_T_, μ_N_ are assumed to have a trivariate normal distribution with a mean vector zero. It has a covariance matrix of the form:

(5) $$\text{cov }{\left({\text{μ}}_{\text{T}}\text{, }{\text{μ}}_{\text{N}}\text{, ε }\right)}^{\text{N}}=\Sigma =\left[\begin{array}{ccc} \sigma_{T}^{2} & \sigma_{TN} & \sigma_{T\varepsilon }\\ \sigma_{TN} & \sigma_{N}^{2} & \sigma_{N\varepsilon }\\ \sigma_{T\varepsilon } & \sigma_{N\varepsilon } & \sigma_{\varepsilon }^{2} \end{array}\right]$$

where

var(μ_T_) = σ_T_^2^, var(μ_N_) = σ_N_^2^, var(ε) = σ_ε_^2^, cov(μ_T_, μ_T_) = σ_TN_, cov(μ_T_, ε) = σ_Tε_ and (μ_N_, ε) = σ_Nε_^'^

Due to this, the error terms in Eq. (4), conditional on the sample selection criterion, have non zero expected values, and the OLS estimates of coefficients β_T_ and β_N_ suffer from sample selection bias (Lee, 1982).

The expected values of the truncated error terms thus (μ_T_ |D= 1) and (μ_N_ |D= 0), according to (Balogun, 2007) are of the form:

Ε (μ_N_ |D = 0) = Ε (μ_N_ |ε ≤ - Ζ'γ)

(6) $$\sigma_{N\varepsilon }\frac{-\varphi ({Ζ}^{\prime }\gamma |\sigma )}{1-Ф({Ζ}^{\prime }\gamma |\sigma )}\equiv \sigma_{N\varepsilon }\lambda_{N}$$

and

Ε (μ_T_ | D = 1) = Ε (μ_T_ |ε > - Ζ'γ)

(7) $${\text{= σ}}_{\text{Tε}}\frac{\text{φ}\left({\text{Ζ}}^{\text{′}}\text{γ}/\text{σ}\right)}{\text{Ф}\left({\text{Ζ}}^{\text{′}}\text{γ}/\text{σ}\right)}={\text{σ}}_{\text{Tε}}{\text{λ}}_{\text{T}}$$

The cumulative distribution function and probability density of the standard normal distribution are Ф and φ, respectively. The ratio of φ and Ф measured at ${Ζ}^{\prime }\gamma$ is termed as the inverse Mills ratio $\lambda_{T\;},\;\gamma_{N}$ (i.e. selectivity terms). The incorporation of selectivity terms into Eq. (4) helps in dealing with the selection bias.

The estimation model is carried out in two separate stages. The first stage estimation is carried out by using probit regression model to analyse the probability of transfer. Its estimation parameter γ is captured in Eq. (3). The estimates obtained are used for the calculation of selectivity term $\lambda_{T\;,}\;\lambda_{N}$ based on Eqs. (6) and (7). The problem of heteroskedasticity is a challenge associated with the two-step approach as it produces residuals that are heteroskedastic (Lokshin and Sajaia, 2004).

To help deal with the shortcoming associated with the use of two-step approach, full information maximum likelihood approach proposed by Lokshin and Sajaia (2004) is adopted since it has the ability to simultaneously handle the transfer decision and outcome equations. The signs and significance levels of the correlation coefficients (*ρ*) estimates are of great importance. Having either $\rho_{T\varepsilon }\left(\sigma_{T\varepsilon }/\sigma_{T}\sigma_{\varepsilon }\right)$ or $\rho_{N\varepsilon }\left(\sigma_{N\varepsilon }/\sigma_{T\varepsilon }\right)$ significantly different from zero is an indication of endogenous switching and can lead to selection bias. *ρ* > 0 is an indication of negative selection bias, meaning landlords with below average income are more likely to transfer their farmlands. Also, *ρ* < 0 shows positive selection bias, meaning landlords with income above average are more likely to transfer their farmlands.

The objective of this study is to analyse the effect of land transfer on landlords' income. This is achieved by first specifying the expected values of the outcomes as suggested by (Fuglie and Bosch, 1995; Abdulai and Huffman, 2014). The landlord who has transferred his/her farmland with characteristics х and Ζ, the expected value of income, Y_jT_, is given as

(8) Ε(Y_jT_ | D = 1) = х*β*_jT_ - σ_Tε_λ_T_

The last term takes into account the issue of sample selection, indicating that landlords that have transferred their farmlands may behave differently from an average landlord with identical characteristics attributed to unobserved factors (Maddala, 1986). The expected value of the same landlord if he had chosen not to transfer his/her farmland is given as

(9) Ε (Y_jN_| D = 1) = X*β*_jN_ - σ_Nε_λ_T_

The difference in income due to transfer is the difference between landlords who have transferred their farmlands and those who have not transferred. Eqs. (8) and (9) are used to obtain unbiased estimates of transfer effects. They produce average treatment effects (ATT) results (Lokshin and Sajaia, 2004; Abdulai and Huffman, 2014) and shown as:

(10) $$ATT=\;Ε\left(Y_{jT}{|}_{D}=1\right)-Ε\left(Y_{jN}{|}_{D}=1\right)=X\left(\beta_{jT}-\beta_{jN}\right)+\;\left(\sigma_{T\varepsilon }-\;\sigma_{N\varepsilon }\right)\;\lambda_{T}$$

where σ and λ represent covariance of the error term and inverse Mills ratio, respectively. The estimation of the outcome looks at the income of landlords. It is important to note that if comparative advantage is the criterion for self-selection, then $\sigma_{T\varepsilon }-\;\sigma_{N\varepsilon }$ would be positive implying that farmland transfer would lead to higher incomes than under random selection (Maddala, 1986).

To enable us cater for endogeneity problems, we adopted the approach recommended by Rivers and Vuong (1988), as the dependent variable is in binary form. The estimation is done by specifying the potential endogenous variables as a function of all the independent variables in the transfer equation with a set of instruments in the first-stage regression inclusive.

The issue of endogeneity may arise in the transfer specification with variables such as party membership and public infrastructure. It is assumed that party membership is a form of assurance in times of need and will directly influence transfer decision but may not have impact on income. Therefore, landlords who are party members can easily transfer their farmlands compared to non party members due to assurance that the former has in times of need. We also assume that farmland transfer depends largely on availability of tenants. Nevertheless, tenants consider key public infrastructure in a particular jurisdiction before renting land. We, therefore, argue that availability of public infrastructure will improve farmland transfer market and hence lands that would have been lying idle as result of no tenants will now have tenants to rent. In addition, the presence of tenants, which is facilitated by the presence of public infrastructure, will influence landlords who initially would have not made up their mind to engage in farmland transfer. In this study, we assume that availability of public infrastructure will influence farmland transfer but will not have influence on income.

Party membership and public infrastructure may be jointly influenced the decision to transfer in the transfer specification. The specification is given as:

(11) $$T_{i}=\gamma {Ζ}_{ij}+\;\psi V_{ij}+\;\zeta_{ij}$$

Where T_i_ denotes the vector of the endogenous variables (party membership and public infrastructure), Ѵ_ij_ denotes the vector of instruments that is correlated with a given endogenous variable but not correlated with the error term ε_j_ in Eq. (3) and eliminated from Eq. (3) estimation. Ζ is explained previously. Instead of relying on the predicted estimates from the first-stage equation as in a usual two-stage estimation approach, the method involves specifying the transfer equation as shown in Eq. (3) as

(12) D_ij_ ∗ = *β*Ζ_ij_ + φT_i_ + R_ij_ + μ_ij_

where Ζ_ij_ is a formerly defined, T_i_ is the vector of the potential endogenous variables (party membership and public infrastructure) and the vector of the residual terms is denoted by R_ij_ from the first-stage regression involving endogenous variables.

The estimates of the potential endogenous variables through the use of probit model is consistent with Wooldridge (2016). Some of the variables in the first-stage estimation in Eq. (11) are eliminated from the transfer equation in (12) in order to ensure the identification in the estimation of the transfer specification. A more appropriate step in the identification is to use a variable that does not influence the transfer decision but has an influence on the endogenous variable. The Table 1 below presents the variables under consideration, their mode of measurement and a prior expectations.

**Table 1:** Variables and their mode of measurement.

Variable	Mode of measurement	A prior expectation
Land transfer	1 yes, 0 otherwise	
Gender	1 male, 0 otherwise	−/+
Age	Number of years	−/+
Per capita land (size)	Number of acreage	+
Off-farm income (RMB)	Amount (RMB)	+
Credit access	1 yes if landlord is able to access credit, 0 otherwise	+/-
Communist Party Member	1 for yes, 0 otherwise	+
Education	Number of years	+/-
Famine experience	1 for yes if a household has in the past experienced an extreme crisis of access to adequate food or currently experiencing extreme crisis of access to adequate food, 0 otherwise	-
Public infrastructure	1 for yes, 0 otherwise	+/-
Skill training	1 for yes, 0 otherwise	+
Out-migration	1 yes if at least a household member has migrated, otherwise	+
Remittance	1 if receives remittance, 0 otherwise	+
**$1 = 6.996RMB**		

Table 2 presents results on descriptive statistics. Minimum and maximum annual incomes of landlords are 1,972.00 RMB and 450,000.00 RMB, respectively. Annual average income of landlords is 17385.83 RMB. Minimum and maximum ages of landlords are 23 and 87 years, respectively, with an average age of 49 years, which is an indication that most of the landlords are within the active population. Off-farm income of the landlords is 6,000.00 RMB minimum and 23,500.00 RMB maximum. An average off-farm income of landlords is 8,538.52 RMB. The minimum and maximum per capita land is one and four acreage, respectively. Average per capita land is approximately two-acreage.

**Table 2:** Summary statistics.

Variable	Observations	Mean	Std. Dev.	Min	Max
Income (RMB)	260	17385.83	31817.03	1972	450000
Out-migration	260	0.330769	0.471398	0	1
Gender	260	0.488462	0.500831	0	1
Famine experience	260	0.307692	0.462429	0	1
Public infrastructure	260	0.919231	0.273006	0	1
Credit access	260	0.546154	0.498826	0	1
Age	260	49.06923	13.64876	23	87
Education	260	9.353846	6.101284	0	26
Party membership	260	0.015385	0.123314	0	1
Per capita land	260	1.65	0.773973	1	4
Transferred land	259	0.625483	0.484935	0	1
Off-farm income (RMB)	260	8538.52	7663.23	6000	23500
Skill training	260	0.557692	0.771437	0	1
Remittance	260	0.5	0.972598	0	1
**$1 = 6.996RMB**					

## 4 Empirical results

Table 3 presents the results on factors influencing the farmland transfer decision and the impact of transfer on landlords' income. Transfer and income equations are jointly estimated using full information maximum likelihood approach. The result on the factors influencing the transfer of land, thus the selection is shown in the second column of Table 3. The correlation coefficients rho_1 and rho_2 are positive and negative, respectively, and significant for both. Since rho_1 is positive and significantly different from zero the model suggests that landlords who choose to transfer their farmlands will earn more income than those who have not transferred. The variables sigma, /lns1, lns2, /r1, and /r2 are ancillary parameters used in the maximum likelihood procedure. Sigma1 and Sigma 2 are the square roots of the variances of the residuals of the regression part of the model and lnsig is its log. r1 and r2 are the transformation of the correlation between the errors from the two equations.

**Table 3:** Endogenous switching regression results for transfer and impact of transfer on income.

Variables	Outcome					
	Selection	Std. Dev.	Transferred	Std. Dev.	Non-transferred	Std. Dev.
Constant	0.843	0.580	5.746∗∗∗	1.256	1.503	1.585
Out-migration	0.158∗∗∗	0.030	0.334∗∗∗	0.070	-0.419∗∗∗	0.106
Famine experience	-1.029∗∗∗	0.127	0.418	0.328	-0.185	0.433
Off-farm income	0.260∗∗	0.102	0.565∗∗∗	0.200	0.696∗∗	0.285
Credit access	-0.151∗∗	0.045	0.122∗∗∗	0.014	0.417∗∗∗	0.094
Age	-0.061∗∗∗	0.006	-0.540∗∗∗	0.113	-0.007	0.018
Agesq	0.083∗∗	0.036	0.342∗∗	0.152	-0.370	0.268
Skills training	0.527∗∗∗	0.083	0.245∗∗	0.103	0.180∗∗	0.073
Education	-0.014∗∗∗	0.002	0.035	0.041	0.010	0.039
Remittance	0.802∗∗∗	0.127	0.961∗∗	0.363	1.373∗∗∗	0.242
Per capita land	-0.132	0.102	-0.328	0.214	-0.122	0.260
Gender	-0.200	0.150	0.106	0.329	0.775	0.406
Public infrastructure	0.169∗∗	0.081				
Party Membership	-0.013	0.026				
/lns1	0.844∗∗∗	0.069				
/lns2	0.887∗∗∗	0.144				
/r1	2.568∗∗∗	0.382				
/r2	-1.577∗∗∗	0.394				
sigma_1	2.326∗∗∗	0.160				
sigma_2	2.428∗∗∗	0.351				
rho_1	0.988∗∗∗	0.009				
rho_2	-0.918∗∗∗	0.062				
Likelihood ratio test of independent Eq. (1)	16.76∗∗∗					
Log likelihood	-637.26317					
Number of observations	260					
Wald chi2(9)	17.19					
Prob > chi2	0.0458					

Sources: Authors' own analysis; 1% ∗∗∗, 5% ∗∗.

The coefficients are interpreted in the same way as normal probit coefficients. The $\chi^{2}$ statistics shown in Table 3 for the validity tests of the overidentifying fail to reject the exclusion restriction that the instruments employed affect transfer only through party membership.

The result shows that out-migration of at least a household member has a positive effect on landlords' transfer decision and the coefficient statistically different from zero (p < 0.001), an indication that landlords who have at least a member of the household migrating to the urban areas for employment are more likely to transfer their farmland. This result partly supports the study by Su et al. (2018), which indicated that willingness to migrate to urban areas has a positive influence on farmland rentals. It further corroborates the study by Chen et al. (2010), which found that households with members migrating to urban areas are more likely to transfer their farmlands to tenants compared to those without members migrating. This result in part reveals the importance of urban and rural interaction as means to generating opportunities for rural dwellers as a form of motivation for farmland transfer (Udimal et al., 2018). It further supports the study by Che (2016a) who found that households with members participating in off-farm work or migrating to the urban cities are more likely to engage in rent out. The result on famine experience, which measured whether a household has in the past experienced an extreme crisis of access to adequate food or currently experiencing an extreme crisis of access to adequate food, shows a negative relationship with farmland transfer. The famine experience variable has a negative influence on landlords' farmland transfer decision. This result confirms the assertion that the past influences individual current decision-making. The finding corroborates the study by Deng et al. (2019), which indicated that households who have had famine experience tend not to rent out their farmlands but rather try to acquire more land (rent in). The findings further corroborate researches by (Cavicchioli et al., 2018; Zou et al., 2018), which asserted that farmland transfer is not influenced solely by economic factors but also historical experience of landlords. Studies by (Almond et al., 2007; Chen and Zhou, 2007; Meng and Qian, 2009) noted that households, which have had famine experience as less, motivated to move to off-farm livelihood activities but rather try to acquire more land (rent in) the assertion which has been corroborated by the study.

The result shows that off-farm activities influence farmland transfer positively. This is statistically different from zero. This suggest that, landlords who engage in off-farm income activities are more likely to rent out their farmlands compared to those not engaged in off-farm income activities (Zou et al., 2018).

Access to credit shows whether the landlord has access to credit or not. The result shows that access to credit by landlords has a negative impact on farmland transfer and coefficient is statistically different from zero (p < 0.001), an indication that landlords who have access to credit facilities will not transfer their farmlands to tenants but rather invest in their farmlands. Access to credit facilities offer opportunities to landlords to engage in expansionary activities such as acquiring of modern inputs and renting of more farmland. This refutes finding by Zou et al. (2018), which indicated that direct subsidy to farmers will promote farmland rent out.

The age variable has negative influence on landlords' decision to transfer their farmlands and statistically significant from zero. Landlords within a certain bracket are unwilling to rent out their farmlands. We further accounted for the fact that the impact of age might not be linear for all age groups; age squared has been incorporated in the analysis. The result shows that agesq has a positive impact on land transfer and statistically different from zero. The negative impact of age together with the positive impact of age squared on land transfer, show that the association between age and land transfer is a more quadratic than linear relation ─ the effect of age on land transfer becomes more important as people get older. This finding corroborates the study by Zou et al. (2018), which indicated that older landlords are more likely to rent out their farmlands, especially for those without successors. The result shows that skills training have a positive impact on landlords' farmlands transfer decision. Landlords who have received skills training are more likely to transfer their farmlands compared to those who have not received skills training. Skills training afford landlords the opportunity to engage in off-farm income activities within and outside their communities. The result further shows education and remittance, respectively, have negative and postive influence on farmland transfer decision and statistically different from zero.

The results on the impact of farmland transfer on landlords' income for the transferred and non-transferred landlords is shown in the column 3 and 4 of Table 3, respectively. As noted earlier, to ensure the identification of the model it is required that at least one variable in the transfer equation or selection equation is excluded from the outcome equation. Party membership and public infrastructure supply are used as identification instruments. They are expected to influence landlords' farmlands transfer decision. They will however, not directly influence landlords' income. The result shows that out-migration contributes positively to transferred landlords' income, it was however not significant for non-transferred landlords. The result shows that off-farm activities contribute positively to both transferred and non-transferred landlords income and statistically different from zero. The result shows that having access to credit improves both transferred and non-transferred landlords' income. Having access to credit is beneficial to both transferred and non-transferred landlords as it contributes positively to their respective incomes. Age of the landlord has a negative effect on the income of landlords who have transferred their farmlands and the coefficient is statistically different from zero. At certain age, it is more profitable to keep ones farmland than transferring especially when there are no opportunities for one to engage in after transfer. However, agesq shows a positive impact on income of landlords who have transferred their farmlands and statistically different from zero. At certain age, it is worth transferring farmland than keeping it, as one cannot engage in any meaningful farming activities. This is an indication that the relationship between age and income is non-linear. The result shows that receiving remittances has positive and significant effect on both transferred landlords and non-transferred landlords' income and statistically different from zero. Remittance adds to the income of landlords hence the positive relationship.

The results further show that skill training has positive relationship with income for both landlords who have transferred and those who have not transferred their farmlands. This is statistically different from zero. Skills training opens array of opportunities for job seekers both within and outside one's immediate environment, and reduces the tendency of idling. Skills training provide opportunity for landlords to add to their flow of incomes by engaging in other income opportunities.

Table 4 above presents the result on average treatments effects (ATT). It measures the impact of farmland transfer on landlords' income. The bias normally arising from the selection of transferred landlords and non-transferred landlords, which may be systematically different is accounted by average treatment effects estimate. The result shows that farmland transfer leads to an increase in landlords' income and coefficient is statistically different from zero. The transfer effect of farmland on landlords' income is approximately 19% increase in annual income. This means if income is the main objective of landlords, transferring their farmlands will be more beneficial as they stand to gain from transfer compared to them farming on their own.

**Table 4:** Impact of land transfer on landlords' income.

	Mean Outcome		ATT	*t*-value
	Transferred	Non-transferred		
Annual Income	26.75	22.50	4.25∗∗∗	6.45

Note: ATT, average treatment effect on the treated.

## 5 Conclusion

This paper uses household level data obtained from landlords to examine factors that influence farmland transfer decision, and also assess the impact of farmland transfer on landlords' income in Xundian County of Yunnan Province.

Endogenous switching regression model was used for the analysis as it is able to account for selection bias, and able to deal with the differential impact of land transfer on transferred and non-transferred landlords. The results revealed that bias associated with sample selection would have been a problem, if the outcome equation was not analysed to cater for the transfer decision. From the result, it was shown that positive selection bias was shown for income, an indication that landlords who have alternative sources of livelihood are more likely to transfer their farmlands.

Factors such as off-farm income, remittance, out-migration, agesq, skills training and public infrastructure has a positive influence on farmland transfer decision. However, famine experience, credit access, education, and age negatively influence farmland transfer decision.

Out migration, off-farm income, credit access agesq and remittance have positive impact on the income of transferred landlords whiles age influences transferred landlords' income negatively. For non-transferred landlords, factors such as off-farm income, credit access skills training and remittances impact positively on their income whilst out-migration influences their income negatively. Farmland transfer impacts positively on landlords' income.

The study brings to light factors that influence farmland transfer and how farmland transfer impacts on landlords' income. Based on this outcome, we recommend the following for policy consideration:

(1) Skill training should be encouraged to offer landlords the opportunity to take up jobs outside the main stream agriculture.
(2) Policies to help out-migrants proper integration in the host cities should be implemented as it will enable out-migrants or their households to transfer their farmlands if they are sure of the sustainability of prospects in the host cities.
(3) Improvement of farmland transfer market should be given policy consideration as it will help improve the economic interest of landlords who are unwilling to transfer their farmlands especially those who have had famine experience and food insecurity.

## Declarations

### Author contribution statement

T.B. Udimal: Conceived and designed the experiments; Analyzed and interpreted the data; Wrote the paper.

E. Liu: Performed the experiments; Analyzed and interpreted the data.

M. Luo: Conceived and designed the experiments; Performed the experiments.

Y. Li: Analyzed and interpreted the data; Wrote the paper.

### Funding statement

This work was supported by 10.13039/501100007846Yunnan Provincial Department of Education Science Research Fund (Grant No. 2019J0199).

### Competing interest statement

The authors declare no conflict of interest.

### Additional information

No additional information is available for this paper.
